# Chromosome level high-density integrated genetic maps improve the *Pyrus bretschneideri* ‘DangshanSuli’ v1.0 genome

**DOI:** 10.1186/s12864-018-5224-6

**Published:** 2018-11-21

**Authors:** Huabai Xue, Suke Wang, Jia-Long Yao, Cecilia H. Deng, Long Wang, Yanli Su, Huirong Zhang, Huangkai Zhou, Minshan Sun, Xiugen Li, Jian Yang

**Affiliations:** 1 Zhengzhou Fruit Research Institute, Chinese Academy of Agricultural Sciences (CAAS), Key Laboratory of Fruit Breeding Technology of Ministry of Agriculture, Zhengzhou, 450009 China; 2 The New Zealand Institute for Plant and Food Research Limited, Auckland, 1025 New Zealand; 3 Guangzhou Gene Denovo Biotechnology Co., Ltd, Guangzhou, 510320 China

**Keywords:** Pear, GBS, Genetic map, SNP, QTL-seq

## Abstract

**Background:**

Chromosomal level reference genomes provide a crucial foundation for genomics research such as genome-wide association studies (GWAS) and whole genome selection. The chromosomal-level sequences of both the European (*Pyrus communis*) and Chinese (*P. bretschneideri*) pear genomes have not been published in public databases so far.

**Results:**

To anchor the scaffolds of *P. bretschneideri* ‘DangshanSuli’ (DS) v1.0 genome into pseudo-chromosomes, two genetic maps (MH and YM maps) were constructed using half sibling populations of Chinese pear crosses, ‘Mantianhong’ (MTH) × ‘Hongxiangsu’ (HXS) and ‘Yuluxiang’ (YLX) × MTH, from 345 and 162 seedlings, respectively, which were prepared for SNP discovery using genotyping-by-sequencing (GBS) technology. The MH and YM maps, each with 17 linkage groups (LGs), were constructed from 2606 and 2489 SNP markers and spanned 1847 and 1668 cM, respectively, with average marker intervals of 0.7. The two maps were further merged with a previously published genetic map (BD) based on the cross ‘Bayuehong’ (BYH) × ‘Dangshansuli’ (DS) to build a new integrated MH-YM-BD map. By using 7757 markers located on the integrated MH-YM-BD map, 898 scaffolds (400.57 Mb) of the DS v1.0 assembly were successfully anchored into 17 pseudo-chromosomes, accounting for 78.8% of the assembled genome size. About 88.31% of them (793 scaffolds) were directionally anchored with two or more markers on the pseudo-chromosomes. Furthermore, the errors in each pseudo-chromosome (especially 1, 5, 7 and 11) were manually corrected and pseudo-chromosomes 1, 5 and 7 were extended by adding 19, 12 and 14 scaffolds respectively in the newly constructed DS v1.1 genome. Synteny analyses revealed that the DS v1.1 genome had high collinearity with the apple genome, and the homologous fragments between pseudo-chromosomes were similar to those found in previous studies. Moreover, the red-skin trait of Asian pear was mapped to an identical locus as identified previously.

**Conclusions:**

The accuracy of DS v1.1 genome was improved by using larger mapping populations and merged genetic map. With more than 400 MB anchored to 17 pseudo-chromosomes, the new DS v1.1 genome provides a critical tool that is essential for studies of pear genetics, genomics and molecular breeding.

**Electronic supplementary material:**

The online version of this article (10.1186/s12864-018-5224-6) contains supplementary material, which is available to authorized users.

## Background

Pear (*Pyrus* spp.) is an important temperate fruit crop that is popular worldwide because of its sweet and juicy flesh, excellent eating quality, and nutritional, medicinal and economic value [1, 2]. In the ancient Chinese pharmacopoeia *Compendium of Materia Medica* (1596), pears were thought to resolve phlegm and relieve cough and asthma. In 2016, the area used for pear cultivation in China exceeded 1.113 million ha and yielded a harvest of 18.70 million tons of fruit (National Bureau of Statistics of the PRC, <http://data.stats.gov.cn/search.htm?s=%E6%A2%A8>, September 26, 2018, data last accessed), which accounted for more than 70% of global pear production (FAOSTAT, <http://www.fao.org/faostat/en/#data/QC>, September 26, 2018, data last accessed).

At present, conventional hybrid breeding is still the main method for generating new pear cultivars. However, the efficiency of this method is low in pear, because of its large tree size, long juvenile phase, and high genome heterozygosity [3–5]. The tens of thousands of hybrid trees required for breeding occupy a large area of land for many years and consume significant manpower and financial resources. Molecular marker-assisted selection (MAS) based on Quantitative Trait Locus (QTL) mapping of important agronomic traits allows breeders to screen hybrids at the seedling stage and eliminate those lacking potential [4, 6]. Thus, this practice can reduce the land, labor and financial investment required for crop breeding, especially for perennial fruit trees [4]. Developing molecular markers linked to important agronomic traits is an important part of molecular breeding.

Significant progress has been made in developing and applying genetic markers in pears over the last two decades. Iketani et al. constructed two genetic maps from the segregation data of 82 F1 individuals and mapped the resistance allele of pear scab and the susceptibility allele of black spot in different linkage groups of ‘Kinchaku’ in 2001 [7]. Since then, researchers have used RAPD, SRAP, AFLP, SSR and other molecular markers to construct pear genetic maps and identified QTLs for agronomic traits [8–12]. In recent years, a hybrid population of ‘Bayuehong (BYH)’ and ‘Dangshansuli (DS)’, which was established by the Zhengzhou Fruit Research Institute of the Chinese Academy of Agricultural Sciences, has been used by several research teams to construct genetic maps and map QTLs for dozens of fruit traits, including fruit weight, fruit shape index, soluble solid content, and fruit maturity [3, 5, 6, 13–16].

Genetic maps constructed using RAPD, AFLP and SSR markers are not ideal for map-based cloning or identification of genes responsible for important traits because of the low density of the mapped markers. Following the application of next generation sequencing (NGS) technology, high density pear genetic maps constructed with SNP markers were used for QTL mapping and map-based gene cloning [6, 17, 18]. For example, Yao et al. [18] identified the *MYB114* gene responsible for the red skin trait using a high-density BYH × DS (BD) map [3]. Furthermore, high-density genetic maps can guide the anchoring of scaffolds to pseudo-chromosomes. Wu et al. anchored 796 scaffolds of 386.7 Mb (or 75.5%) of DS v1.0 genome sequences to 17 pseudo-chromosomes according to a genetic map consisting of 2005 SNP markers [1]. Chagné et al. anchored 868 scaffolds of 171.3 Mb (29.7% of the genome) to the 17 pseudo-chromosomes of *Pyrus communis* ‘Bartlett’ according to an integrated map consisting of 2279 markers (1391 and 888 apple and pear SNPs, respectively, genetically mapped using seven apple full-sib families and five pear segregating populations) [2, 19, 20]. Recently, Li et al. [6] anchored another 291.5 Mb of the ‘Bartlett’ genome scaffolds in 17 pseudo-chromosomes using three high-density SNP genetic maps (OH × LBJ, BYH × DS-JXB and PH-CG) with European pear genetic backgrounds, which dramatically increased the anchored portion of the ‘Bartlett’ genome from 29.7% in the original assembly to 50.5%. The assembled genomes of DS and Bartlett provide useful tools for fine mapping of genes/QTLs and map-based gene cloning, and they have laid a solid foundation for future pear genetics and genomics research. However, pseudo-chromosome genome assemblies do not yet exist for ‘Bartlett’ or DS.

Scaffold anchoring of the two published pear genomes was performed using genetic maps constructed with F1 populations that were relatively small in size, which may have resulted in errors and conflicts, some of which were discovered in subsequent analysis [6, 21]. High-density consensus genetic maps constructed by integrating high-quality genetic maps have been used to improve the quality of pear genome assemblies [6]. In this study, we used two relatively large F1 populations of Asian pear to construct genetic maps. By integrating these two new maps with the BD map [3], we were able to improve the scaffolding of the DS reference genome v1.1 to the chromosome level <http://genedenovoweb.ticp.net:81/pear/>.

## Results

### Construction of new genetic maps for MTH × HXS and YLX × MTH

A total of 778 and 338 million clean reads were obtained from the MTH × HXS and YLX × MTH populations, yielding more than 92.9 Gb and 46.3 Gb of clean data, respectively. The number of reads per individual plant varied from 0.88 to 3.56 million reads in MTH × HXS and from 1.63 to 3.45 million reads in YLX × MTH. The reads obtained from the parents were as follows: 10.06 million for MTH, 7.13 million for HXS, and 2.96 million for YLX (Additional file 1: Table S1). From these data, 31,423 putative SNP markers were obtained from MTH × HXS, consisting of 13,259 lmxll (42.2%), 12,345 nnxnp (39.3%) and 5819 hkxhk markers (18.5%), whereas 28,491 SNP markers were obtained from YLX × MTH, consisting of 9552 lmxll (33.5%), 13,388 nnxnp (47.0%), and 5551 hkxhk markers (19.5%). After removing unqualified and redundant SNPs, qualified SNPs for MTH × HXS and YLX × MTH were loaded into JoinMap 4.1. Finally, 2606 and 2489 SNP markers were used for final map construction for the MTH × HXS and YLX × MTH populations, respectively (Additional file 1: Table S2).

The statistical data for the genetic maps of MTH × HXS and YLX × MTH are shown in Table 1. The total length of the MTH × HXS map was 1846.6 cM, with an average distance between markers of 0.7 cM and a LG length ranging from 83.0 to 162.9 cM. The total genetic length of the YLX × MTH map was 1667.9 cM, with an average distance between markers of 0.7 cM and a LG length ranging from 74.7 cM to 141.2 cM. The largest gaps of the MH and YM maps were 15.5 cM in LG16 and 10.1 cM in LG17, respectively. Although the average genetic distance between markers is not the smallest, the numbers of LGs with gaps > 10 cM and > 5 cM in the MH and YM maps were three and ten and one and eleven, respectively, which were far fewer than the corresponding numbers in the BD (> 10 cM: 10, > 5 cM: 17) and RM (> 10 cM: 8, > 5 cM: 16) maps with the highest density of markers based on different pear reference genomes. This finding indicates that the markers on the MH and YM maps were more evenly distributed on the LGs.

**Table 1:** Description of two pear genetic maps

Group ID	MH map				YM map			
	Number of markers	Length (cM)	Average (cM)	Max gap (cM)	Number of markers	Length (cM)	Average (cM)	Max gap (cM)
1	117	107.2	0.9	5.3	117	103.1	0.9	4.5
2	182	96.9	0.5	5.4	145	97.1	0.7	9.4
3	124	102.8	0.8	4.3	150	107.6	0.7	6.6
4	107	83.0	0.8	6.5	136	83.9	0.6	7.0
5	149	101.8	0.7	3.1	142	95.6	0.7	5.0
6	214	162.9	0.8	7.4	190	141.2	0.7	5.9
7	160	109.2	0.7	12.3	122	77.0	0.6	5.2
8	148	113.5	0.8	14.3	136	107.4	0.8	6.0
9	212	122.0	0.6	2.4	179	114.5	0.6	6.0
10	201	123.0	0.6	7.6	165	108.9	0.7	4.4
11	111	92.7	0.8	3.1	141	94.1	0.7	3.8
12	122	83.2	0.7	3.1	145	74.7	0.5	3.5
13	182	101.0	0.6	4.3	170	81.5	0.5	3.3
14	127	115.0	0.9	6.4	134	83.3	0.6	5.9
15	156	104.0	0.7	8.0	122	86.8	0.7	5.5
16	117	122.4	1.0	15.5	159	101.8	0.6	8.4
17	177	105.8	0.6	4.6	136	109.3	0.8	10.1
all	2606	1847	0.7	15.5	2489	1668	0.7	10.1

### Integration of three genetic maps and anchoring scaffolds into the integrated map

**Fig. 1:**
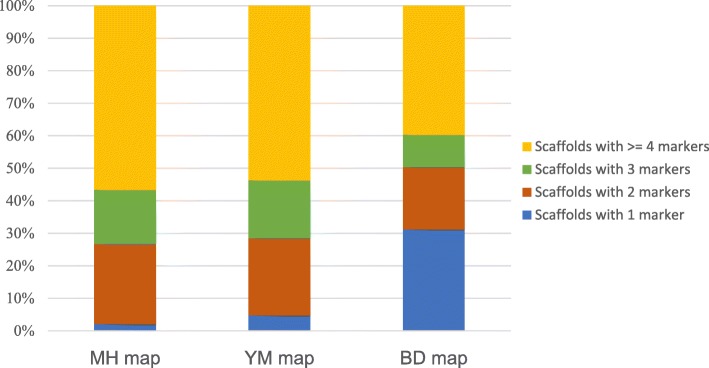
Proportion of scaffolds with different numbers of markers in each of the three genetic maps

The DS reference genome scaffolds were anchored to the three genetic maps (Table 2). 2606, 2489 and 3143 unique markers from the MH, YM, and BD maps, corresponding to an average physical marker density of 7.4 markers/Mb, 7 markers/Mb, and 9.3 markers/Mb, respectively, were loaded into ALLMAPS. Although the BD map had the highest marker density, the difference in the number of scaffolds anchored among the three maps was very small, and the proportions of the scaffolds of the MH and YM maps that covered the total genome length were even larger than that of the BD map. The percentage of scaffolds anchored with only one marker was 2.09, 4.77 and 31.22% for MH, YM and BD, respectively, while 97.91, 95.23 and 68.78% of the scaffolds anchored in the MH, YM and BD maps had two or more markers, respectively. This finding confirms that the markers in the MH and YM maps are more evenly distributed across the genome in comparison with the markers in the BD map (Fig. 1).

**Table 2:** Summary of the genome coverage of each individual map

Map	MH map	YM map	BD map
Linkage Groups	17	17	17
Markers (unique)	2606	2489	3143
Markers per Mb	7.4	7	9.3
N50 Scaffolds	246	249	232
Scaffolds	670	671	679
Scaffolds with 1 marker	14	32	212
Scaffolds with 2 marker	165	159	130
Scaffolds with 3 marker	112	120	68
Scaffolds with > = 4 marker	379	360	269
Total bases	351,322,567	353,618,398	336,225,218
Percentage of genome size	69.10%	69.50%	66.10%

The MH, YM, and BD genetic maps were integrated to form a consensus MH-YM-BD map using ALLMAPS (Table 3 & Table 4 for a summary, Additional file 1: Table S1 for a detailed list). The consensus map contained a total of 7863 unique markers. With 7757 markers, 898 scaffolds were anchored into 17 pseudo-chromosomes. Scaffolds containing the rest 106 markers, were not anchored into pear pseudo-chromosomes due to a lack of markers or positional conflicts. The anchored scaffolds accounted for 78.8% of the assembled genome size. Of these, 793 scaffolds (88.3% of the anchored scaffolds), making up 74.5% of the total genome length, were anchored with two or more markers, so that it was also possible to determine their orientation on the chromosomes. The unanchored 1284 short scaffolds accounted for only 21.20% of the assembled genome length. The DS genome v1.1 chromosome level sequences in FASTA format are available at our Pear Genomics & Breeding website <http://genedenovoweb.ticp.net:81/pear/>.

**Table 3:** Summary of the consensus map

	Anchored	Oriented	Not anchored
Markers (unique)	7757	7455	106
Markers per Mb	19.4	19.7	0.9
N50 Scaffolds	263	256	14
Total number of scaffolds	898	793	1284
Scaffolds with 1 marker	73	0	16
Scaffolds with 2 marker	104	96	9
Scaffolds with 3 marker	93	89	2
Scaffolds with > = 4 marker	628	608	9
Average size of each scaffolds (bp)	446,075	477,594	84,093
Total bases	400,575,263	378,731,939	107,975,332
Percentage of genome	78.80%	74.50%	21.20%

**Table 4:** Summary of the DS v1.1 genome assembly

Pseudo-chromosome	Length of pseudo-chromosome (bp)	Number of scaffolds	Length of scaffolds (bp)	Scaffolds N50 (bp)	Markers existing in MH	Markers existing in YM	Markers existing in BD
Chr1	17,744,726	38	17,741,026	542,833	103	137	10
Chr2	21,240,122	46	21,235,622	656,779	149	142	226
Chr3	26,910,613	54	26,905,313	608,190	106	160	192
Chr4	22,563,200	57	22,557,600	526,445	178	144	106
Chr5	26,925,062	60	26,919,162	802,350	124	148	249
Chr6	20,812,769	42	20,808,669	711,089	184	169	195
Chr7	23,078,416	51	23,073,416	713,375	154	122	185
Chr8	17,217,004	39	17,213,204	680,433	161	119	158
Chr9	22,547,832	48	22,543,132	791,334	141	138	173
Chr10	26,043,940	64	26,037,640	546,897	194	159	206
Chr11	31,670,729	75	31,663,329	773,816	211	178	226
Chr12	20,531,346	47	20,526,746	553,039	113	142	151
Chr13	19,244,441	42	19,240,341	737,203	118	112	146
Chr14	21,939,595	60	21,933,695	803,459	127	130	151
Chr15	39,108,076	71	39,101,076	798,494	215	195	350
Chr16	20,399,442	47	20,394,842	713,471	123	134	94
Chr17	22,686,050	57	22,680,450	499,627	179	142	158
Total	400,663,363	898	400,575,263	648,178	2580	2471	2976

### Comparison of the consensus map and the three individual genetic maps

**Fig. 2:**
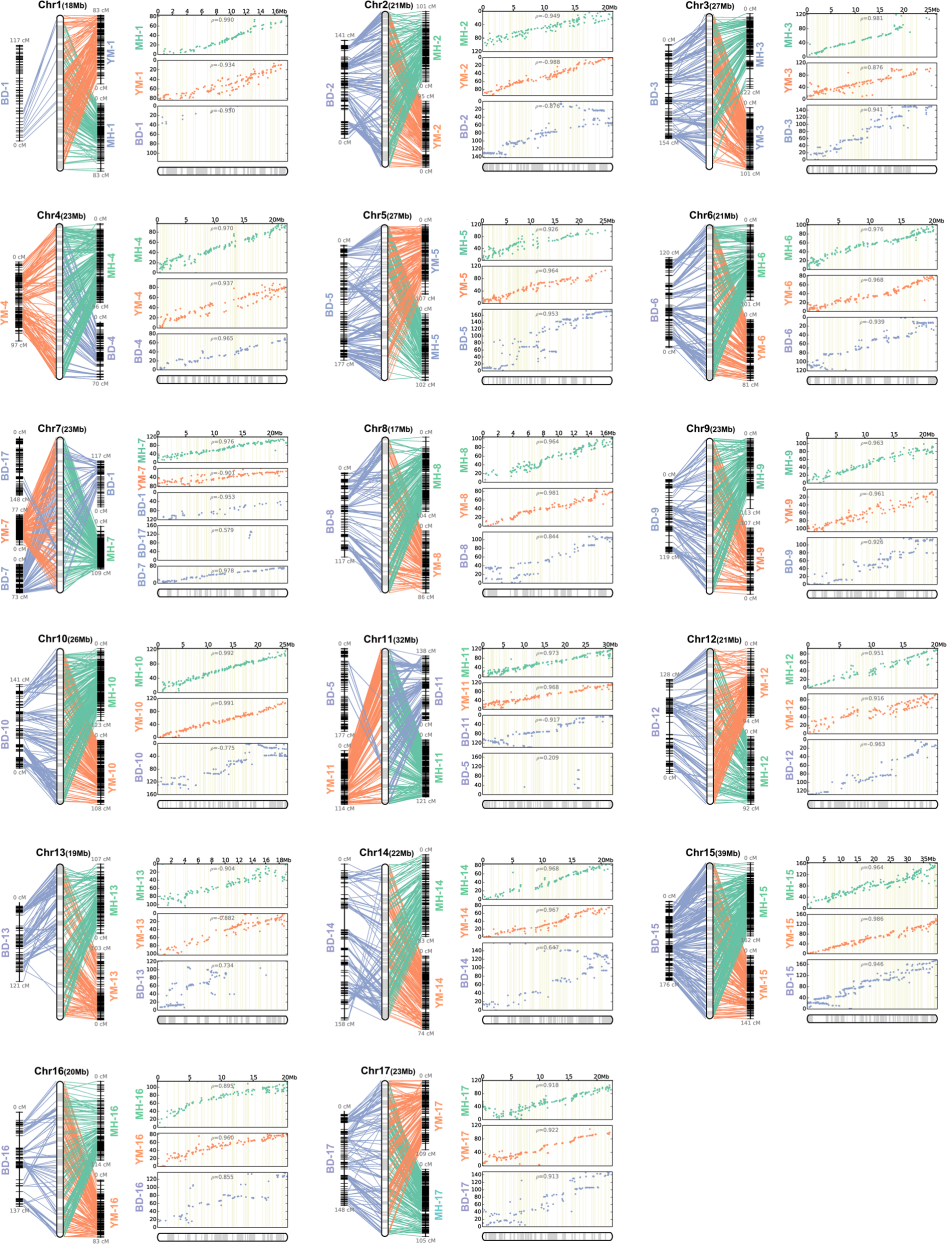
Collinear analysis between the consensus map and the three individual maps. For every pseudo-chromosome, the left figure is a collinear comparison of the consensus map with the three individual maps. The figure on the right shows the consistency of the marker position order between the three individual maps and the consensus map. The X axis is the marker position on the consensus map, and the Y axis is the marker position on the individual maps. The numbers in the figure represent the Spearman rank correlation coefficients between the marker order of the individual maps and the consensus map

ALLMAPS can compute a scaffold ordering that maximizes collinearity across a collection of maps; if there is a conflict within the entered map, ALLMAPS will output the most probable ordering to generate a consistent map. Using the consensus map produced by ALLMAPS as a reference, we compared differences in scaffold order among the three maps and manually checked the errors in each map. For example, LG1 and LG7 (Fig. 2) were not completely separated from each other in the BD map, but they were completely separated in the integrated map, the MH map, and the YM map. LG1 in the BD map was split in two: the smaller part showed collinearity with LG1 of the consensus map, while the larger part, together with LG 7 and a small piece of LG 17, showed collinearity with LG 7 of the consensus map. We therefore integrated the three pieces and re-assigned them to LG7. Furthermore, LGs 1 and 7 in the BD map lacked a long fragment with respect to the consensus map; LG5 in the BD map also looked incomplete, and several parts of LG5 were re-assigned to LG11. According to the MH-YM-BD map, 19, 12 and 14 newly anchored scaffolds were added to Chr1, Chr5 and Chr7, respectively, of the DS v1.1 genome to extend them. Overall, the BD map had a weak correlation with the consensus map, especially on LGs 2, 8, 10, 13, 14 and 16, for which the Spearman rank correlation coefficients to the integrated map LGs were less than 0.9. On the contrary, the MH and YM maps were highly consistent, which helped us to correct the potential error in the BD map and construct a more reliable set of pseudo-chromosomes for the DS v1.1 genome.

### Synteny analyses between the DS genome v1.1 and the GDDH13 apple genome

**Fig. 3:**
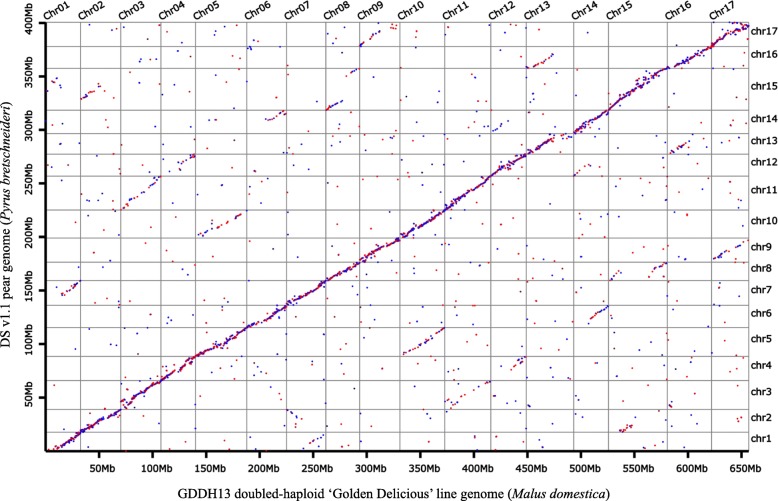
Synteny analyses between the DS v1.1 and the GDDH13 doubled-haploid apple genome

Previous studies have shown that apple and pear have collinear genomes [2, 6]. To further confirm the quality of our newly assembled pear genome, we performed a collinear comparison between the DS genome v1.1 and a previously published GDDH13 apple genome [22]. We used homologous proteins to determine the collinearity of the pear and apple genomes. After Blastp alignment and filtration, 44,840 and 45,110 proteins from pear and apple, respectively, corresponding to 19,454 homologous protein pairs between the two species, were selected and used for the collinearity comparison. There was good one-to-one collinearity between the pseudo-chromosomes of DS genome v1.1 and the GDDH13 apple genome, with high correlation coefficients from 0.9354 to 0.9902 except for pseudo-chromosome 3 (0.8945) (Fig. 3 and Table 5). The overall collinearity between the DS genome v1.1 and the GDDH13 genome was superior to that between the 1^st^ ‘Golden Delicious’ genome and the ‘Bartlett’ genome v1.1 [6], which indicated that the high-quality genetic maps generated in this study improved the accuracy of the pear genome assembly. In addition to the one-to-one correspondence between the pear-apple homologous chromosome pairs, we also observed collinear blocks among non-homologous pear-apple chromosomes: 1–7, 2–7, 2–15, 3–11, 4–12, 5–10, 6–14, 8–15, 9–17, 12–14 and 13–16. This non-homologous inter-chromosomal synteny between species was consistent with the inter-chromosomal synteny within pear and apple [1, 2, 6, 23], which indicated that these genome-wide duplication events occurred in the common ancestor of pear and apple. This finding was consistent with a previous report that whole-genome duplication events in pear and apple occurred earlier than their species differentiation [1].

**Table 5:** Correlation coefficients of pseudo-chromosome pairs between the DS genome v1.1 and the GDDH13 apple genome

Chr	chr1	chr2	chr3	chr4	chr5	chr6	chr7	chr8	chr9	chr10	chr11	chr12	chr13	chr14	chr15	chr16	chr17
Correlation coefficients	0.9721	0.9902	0.8945	0.9705	0.9734	0.9845	0.9868	0.9894	0.9625	0.9816	0.9885	0.9801	0.9396	0.9811	0.9890	0.9773	0.9354

### The relocation of Asian pear red/green locus confirmed the accuracy and convenience of the DS v1.1 genome

78,220,374 reads from the red-skinned DNA pool (19.65× depth coverage or 94.74% coverage) and 93,410,888 reads from the green-skinned DNA pool (22.98× depth coverage or 95.15% coverage) [21] were aligned to the DS v1.1 reference genome and SNPs were identified. After removing low quality SNPs, a total of 4,284,472 SNPs were retained and used to perform the sliding window analysis. The candidate interval of the red/green skin locus was positioned on the fifth chromosome according to the |∆(SNP-index)| plot (Additional file 2: Figure S1), which is consistent with previous results [21]. The candidate interval derived from the threshold line for the top 0.5% in the |∆(SNP-index)| plot was 2.1 Mb, which was separated into two subintervals of 0.9-Mb and 1.2 Mb by a 3.26 Mb gap. The 2.1 Mb interval was comprised of parts of five scaffolds: NW_008988126.1 (779.4 kb, 188,765–968,163 bp of the scaffold), NW_008988141.1 (120.5 kb, 1–120,502 bp of the scaffold), NW_008988091.1 (37.7 kb, 1,156,306–1,193,985 bp of the scaffold), NW_008988243.1 (620.2 kb, 1–620,203 bp of the scaffold) and NW_008988130.1 (542.0 kb, 1–542,018 bp of the scaffold). Four of these five scaffolds were involved in the candidate scaffolds located previously [21], with the exception of scaffold NW_008988243.1.

## Discussion

With the development of high-throughput sequencing technology and the popularity of simplified genomic sequencing technologies such as RAD [24], GBS [25] and SLAF [26], genetic maps with high (or even ultra-high) marker density have laid a foundation for downstream applications such as QTL mapping and chromosome assembly [27–30]. Wu et al. constructed a genetic map of 2005 SNP markers in pear using a BYH × DS cross and anchored scaffolds of the DS genome [1]. Subsequently, they improved the genetic map and identified QTLs by increasing the number of offspring from 56 to 102 and increasing the number of markers to 3241 (the BD map) [3]. Montanari et al. successfully mapped 829 polymorphic pear markers and 569 polymorphic apple markers from their newly developed apple and pear Infinium® II 9 K SNP array to the parental genetic maps for five pear segregating populations, and they found that the number of markers per map varied from 318 to 645 [20]. These parental genetic maps were then used, along with maps for seven apple full-sib families [19], to anchor 171.3 Mb of the assembled ‘Bartlett’ genome into 17 LGs [2]. Recently, Wang et al. built a high-density linkage map with 4865 markers (4664 SNP markers and 201 SSR markers) of the segregating population of ‘Red Clapp’s Favorite’ × ‘Mansoo’ (RM map), spanning 2703.61 cM, with an average distance of 0.56 cM between adjacent markers [31]. To date, the BD and RM maps are the densest pear genetic maps. In this study, we selected only five of the polymorphic parental SNP markers equally distributed on each scaffold to reduce marker redundancy for construction of the MH map and the YM map. This practice did not maximize the number of markers or marker density of these genetic maps, but it led to the generation of maps on which the markers were relatively evenly distributed.

During comparative mapping of multiple genetic maps, some markers may be revealed to be located at different positions of a LG or may even be assigned to different LGs. This phenomenon is observed even between male and female maps from a single outcross population [32], and it may be caused by segregation distortion of markers. Segregation distortion of markers affects the recombination distance between markers and the order of the markers on LGs [33, 34]. Wang et al. found that two distorted markers (NAUpy59t and ZFRIt051) were able to change the order of other markers on a LG [32]. Differences between the genetic backgrounds of parents in different mapping populations can also lead to differences in marker order [31, 32]. In this study, the parent material for the MH map consisted of ‘Mantianhong’ (*P. pyrifolia*) and ‘Hongxiangsu’ (*P. bretschneideri*), whereas the parent material for the YM map consisted of ‘Yuluxiang’ (*P. bretschneideri*) and ‘Mantianhong’, and the genetic backgrounds of the two populations were relatively similar. However, the parents of the BD map were ‘Bayuehong’ (*P. communis*) and DS (*P. bretschneideri*), so their backgrounds were quite different from those of the MH and YM populations [35–38]. Therefore, the markers of the MH and YM maps have relatively good collinearity between each other compared to that of the markers of the BD map. In addition, the presence of incorrect insertions of contigs in some scaffolds [21] and large differences in population size may also be responsible for differences in the order of the markers among the different maps analyzed in this study.

The quality of the genome assembly has an important and direct effect on the anchoring of scaffolds into pseudo-chromosomes [6, 39, 40]. Pootakham et al. constructed an integrated linkage map and anchored 28,965 contigs, covering only 12% of the published rubber tree genome, of which 78% of sequences were identified as repetitive DNA, and the average scaffold length was 1.84 kb [41, 42]. Westbrook et al. mapped 3305 loblolly pine scaffolds onto 12 linkage groups using 3762 markers and found that most scaffolds were too short to span two or more markers [43]. In pear, there were 2103 scaffolds (with an N50 scaffold length of 540.8 kb) and 142,083 scaffolds (with an N50 scaffold length of 88.114 kb) in the DS and ‘Bartlett’ genome assemblies, respectively, however, in comparison with the ‘Bartlett’ genome assembly, more sequences of the DS assembly had been anchored into pseudo-chromosomes with fewer markers [1, 2, 6]. Genetic maps are useful tools for guiding scaffold anchoring into pseudo-chromosome assembly, and the quality and density of genetic maps are important factors [6, 39]. In this study, the polymorphic markers of the parents of two populations were first filtered according to their positions before the genetic map was constructed, so the number of mapped markers in the two genetic maps was not the largest among published genetic maps, but the number of anchored scaffolds of these two maps were both close to that of the previously published BD map, and the total length of the anchored scaffolds in each of these two maps exceeded the total length of the scaffold anchored by the BD map (excluding the SSR markers in the BD map). Moreover, in comparison with the BD map, the direction of more than 95% of anchored scaffolds (anchored with two or more markers) can be confirmed by both newly constructed maps, while the direction of only 68.78% of anchored scaffolds can be determined according to the BD map. This finding indicates that the MH and YM maps had fewer redundant markers and higher quality in comparison with the BD map, and thus they are more useful as guides for pseudo-chromosome assembly.

The new DS v1.1 genome is a greatly improved version in comparison with DS v1.0, with particular improvement to pseudo-chromosomes 1 and 7. In this study, seven scaffolds previously anchored to pseudo-chromosome 1 of DS v1.0 were transferred to pseudo-chromosome 7 of DS v1.1. Of the 38 scaffolds anchored to pseudo-chromosome 1 of DS v1.1, 19 were newly anchored in this study. Only 12 of the other 19 scaffolds were also anchored to pseudo-chromosome 1 according to the BD map, while the other seven were scattered among the other pseudo-chromosomes of DS v1.0. Li et al. reported similar findings in a recent study, in which they integrated existing genetic maps and performed scaffold anchoring. They anchored nine new scaffolds on DS pseudo-chromosome 1 and re-anchored a large number of markers located on LG1 of the BD map (BYH × DS-JXB) to pseudo-chromosome 7 of the DS genome [6]. Five of the nine scaffolds (scaffold 341.0, scaffold 467.0, scaffold 638.0, scaffold 797.0 and scaffold 872.0) newly anchored by Li et al. also appeared in the list of 19 newly anchored scaffolds in this study, which demonstrated the reliability of the DS v1.1 assembly. The partially homologous blocks on pseudo-chromosomes 1 and 7 may be the reason that a large number of markers that should have been mapped onto LG7 in the BD map were instead mapped onto LG1 [1, 6, 23]. However, the failure to anchor more scaffolds onto pseudo-chromosome 1 may also be related to the small size of the population that was used to construct the BD map, as well as lethal genes present in chromosome 1, which can cause interspecific hybrid necrosis [44].

In our previous study, a modified QTL-seq analysis was performed on the red/green fruit skin trait locus of Asian pear at the scaffold level, and the scaffolds linked to the red/green locus were then mapped to LG5 according to the high density BD map [3, 21]. In this study, we directly performed chromosome-level QTL-seq analysis using the new DS v1.1 genome assembly and located the red/green locus in an interval consistent with that reported in previous studies [18, 21], which greatly reduced the workload required to map scaffolds to linkage groups and supported the accuracy of DS v1.1. The peak of the candidate interval in the |∆(SNP-index)| plot (Additional file 2: Figure S1) was split into two parts by a 3.26 Mb gap because most part of each of the three scaffolds (NW_008988076.1, NW_008988141.1 and NW_008988091.1) within the gap were unlinked to the R/G locus [21]. As the three individual genetic maps are highly consistent in the entire region of LG5 (Fig. 2), we were able to rule out the possibility of assembly error regarding the genetic maps. In short, although there were small errors in some scaffolds, the DS v1.1 genome assembly provides a foundation for studies aimed at locating important agronomic traits at the chromosome level.

## Conclusions

In this study, two sets of maps, MH and YM, were constructed and further merged with a previously published BD map to build a new integrative MH-YM-BD map. Eight hundred ninety-eight scaffolds, of which 88.31% were directionally anchored with two or more markers, were then successfully anchored into 17 pseudo-chromosomes of the DS v1.1 genome according to the MH-YM-BD map, accounting for 78.8% (400.57 Mb) of the assembled genome size. Errors in each pseudo-chromosome were corrected. Pseudo-chromosomes 1, 5 and 7 were extended by 50, 20 and 27.5%, respectively. Seven scaffolds from pseudo-chromosome 1 were transferred into pseudo-chromosome 7 in the newly constructed DS v1.1 genome. The DS v1.1 genome, which has high collinearity with the apple genome and was used to accurately locate the red/green locus of Asian pear, provides a critical tool that is essential for studies of pear genetics, genomics and molecular breeding.

## Methods

### Construction of new genetic maps for MTH × HXS and YLX × MTH

Asian pear cultivars ‘Mantianhong’ (MTH) (*P. pyrifolia*), ‘Hongxiangsu’ (HXS) (*P. bretschneideri*) and ‘Yuluxiang’ (YLX) (*P. bretschneideri*) were grown as parental lines in the orchard of the Zhengzhou Fruit Research Institute, Chinese Academy of Agricultural Sciences (ZFRI, CAAS) in Zhengzhou (Henan Province, China). The 345 and 162 hybrid plants resulting from the MTH/HXS cross and YLX/MTH cross, respectively, were used in this study. The hybrids were produced in 2009 and grown at the affiliated experimental orchard of the ZFRI, CAAS in Xinxiang (Henan Province, China). MTH × HXS (MH) and YLX × MTH (YM) genetic maps were constructed using SNP markers derived via genotyping by sequencing (GBS) [25]. Young leaves of each plant were collected in mid-April at the beginning of vegetative growth. The leaves were first frozen in liquid nitrogen and then transferred to a − 80 °C freezer. Total DNA was extracted using the cetyltrimethylammonium bromide method [45], and GBS libraries were constructed using the two-enzyme modification of the original GBS protocol [25, 46]. One hundred ng of DNA for each plant sample was digested with restriction enzymes *EcoR*I and *NIa*III (New England Biolabs, Ipswich, MA, USA). The digested products were then ligated to 25 pmol of A1 and A2 adapters. The libraries were pooled, size-selected (400–600 bp) on a 1% agarose gel, column-cleaned using a PCR purification kit (NEB), and amplified for 12 cycles using Phusion DNA polymerase (NEB). Average fragment size was estimated on a Bioanalyzer 2100 (Agilent, Santa Clara, CA) using a DNA1000 chip following a second column-cleaning. Library quantification was performed using PicoGreen (Invitrogen, Carlsbad, CA, USA). Pooled libraries were adjusted to 10 nmol and sequenced with PE125 on a HiSeq4000 instrument (Illumina, San Diego, CA). High-quality clean reads were obtained by (1) trimming raw reads, (2) removing reads with > 10% unidentified nucleotides and (3) removing reads with > 50% bases having a low Phred quality score (< 5). The Burrows-Wheeler Aligner [47] was used to align the clean reads against the scaffolds of the *P. bretschneideri* DS genome <ftp://ftp.ncbi.nlm.nih.gov/genomes/Pyrus_x_bretschneideri/CHR_Un/> [1] using ‘mem −k 32−M’, where *k* is the minimum seed length and *M* is an option used to mark shorter split alignment hits as secondary alignments. SNP calling was performed on all samples using GATK’s Unified Genotyper 3.3 [48], and SNPs were filtered using GATK’s Variant Filtration with appropriate parameter settings (-Window 4, −filter ‘QD < 4.0||FS > 60.0||MQ < 40.0, −G_filter ‘GQ < 20′). Variants exhibiting segregation distortion or sequencing errors were discarded. The ANNOVAR Software Tool (Philadelphia, PA, USA) [49] was used to annotate SNPs in the genome. Polymorphic parental SNP markers were classified into ‘CP’ population segregation patterns, such as lm × ll, nn × np, and hk × hk, in a Mendelian manner [50]. SNP variants outside the sequencing depth range of 5–1500 were considered missing data. All SNPs with missing data for > 10 individuals were removed from the analysis. Markers showing significantly distorted segregation (Chi-square test, *P* < 0.05), having low integrity (< 95%), or containing abnormal bases were filtered by JoinMap 4.1 [51]. To reduce the number of redundant markers, only five of the SNPs equally distributed on a scaffold were retained for the scaffolds with more than five qualified SNP markers. Next, the qualified SNP markers were used to construct the genetic linkage maps of MH and YM using the Kosambi mapping function in JoinMap 4.1. The LOD value was set to separate most markers into 17 linkage groups (LG), which was consistent with the BYH × DS (BD) map published by Wu et al. [1].

### Construction of high quality pear consensus genetic maps and anchoring of the DS scaffolds

A integrated genetic map for MH, YM and BD was constructed using ALLMAPS software [39]. In the pilot test, we compared the consistency between pairs of maps and found the most consistency between the MH map and YM map. This finding indicated that these two maps had higher reliability in comparison with the other maps. Therefore, for construction of the final integrated map, the weight factor in ALLMAPS was set to 2 for the MH map and YM map, but it was set to 1 for the BD map. The scaffolds were sorted based on the positions of the SNP markers on the three individual maps, after which the scaffolds were integrated to obtain a consensus map. The scaffolds with two or more markers were further defined with regard to their direction in the consensus map, and un-anchored scaffolds were assigned into pseudo-chromosome 0. Consistencies and differences among the three maps (MH, YM and BD) were confirmed by visual evaluation of collinearity.

### Synteny of the new version of the pear genome with the GDDH13 apple genome

We downloaded the GDDH13 doubled-haploid apple genome [22] sequence from NCBI <ftp://ftp.ncbi.nlm.nih.gov/genomes/all/GCA/002/114/115/GCA_002114115.1_ASM211411v1/> and obtained the sequences of protein-coding genes that were homologous to pear genes. These homologous genes were used to search all of the protein sequences of pear and apple using the best reciprocal hit BLAST strategy of the Blastp software (ftp://ftp.ncbi.nlm.nih.gov/blast/executables/blast+), setting the lowest threshold for e < 1 × 10E-7. The one-to-one correspondence between homologous pear and apple chromosomes was determined using the homologous information of each pair of genes. The collinearity diagram for apple and pear was plotted using in house R script based on the physical positions of the homologous genes on homologous chromosomes.

### QTL-seq analysis for the red/green locus of Asian pear to verify the accuracy of the DS v1.1 genome

We directly used previous resequencing data of the red- and green-skinned pools for the QTL-seq analysis. The procedures used for clean read alignment, variant calling, and annotation were the same as those described in a previously published study [21]. Seventeen pseudo-chromosome sequences of the newly assembled DS genome were used as the reference sequences to calculate the SNP-index, Δ(SNP-index) and |Δ(SNP-index)| between the red- and green-skinned pools [21, 52]. Sliding window analysis was performed on the 17 newly assembled DS pseudo-chromosome sequences and applied to the SNP-index and |∆(SNP-index)| plots with 1-Mb windows and 20-kb increments. To avoid the ‘pseudoexchange effect’ in heterozygous crops, |Δ(SNP-index)| was used instead of Δ(SNP-index) as the main parameter to identify the target phenotype [21]. The top 0.5% of the highest |Δ(SNP-index)| intervals were selected as candidate gene intervals [21].

## Additional files

Additional file 1: **Table S1.** Statistics of sequencing data for the crosses MTH × HXS and YLX × MTH. **Table S2.** Details of markers localized in the integrated pear consensus MH-YM-BD map. (XLS 1695 kb)

Additional file 2: **Figure S1.** |Δ(SNP-index)| graph derived from the modified QTL-seq analysis of mapping for the red skin trait of Asian pear. The X-axis indicates the position of the 17 pseudo-chromosomes and the Y-axis indicates the |Δ(SNP-index)|. (PPTX 278 kb)

## Abbreviations

AFLP: Amplified fragment length polymorphism
BD: BYH × DS
BYH: Bayuehong
DS: Dangshansuli
GBS: Genotyping-by-sequencing
HXS: Hongxiangsu
LG: Linkage group
MAS: Marker-assisted selection
MH: MTH × HXS
MTH: Mantianhong
NGS: Next generation sequencing
QTL: Quantitative trait locus
RAPD: Random amplified polymorphic DNA
RM: Red Clapp’s Favorite × Mansoo
SNP: Single nucleotide polymorphism
SRAP: Sequence-related amplified polymorphism
SSR: Simple sequence repeat
YLX: Yuluxiang
YM: YLX × MTH
