# Supplementary figures and images for: Chromosome level high-density integrated genetic maps improve the Pyrus bretschneideri ‘DangshanSuli’ v1.0 genome
Source: BMC Genomics. 2018 Nov 21;19:833. doi: 10.1186/s12864-018-5224-6 (PMC6249763; doi:10.1186/s12864-018-5224-6)

## Slide 1
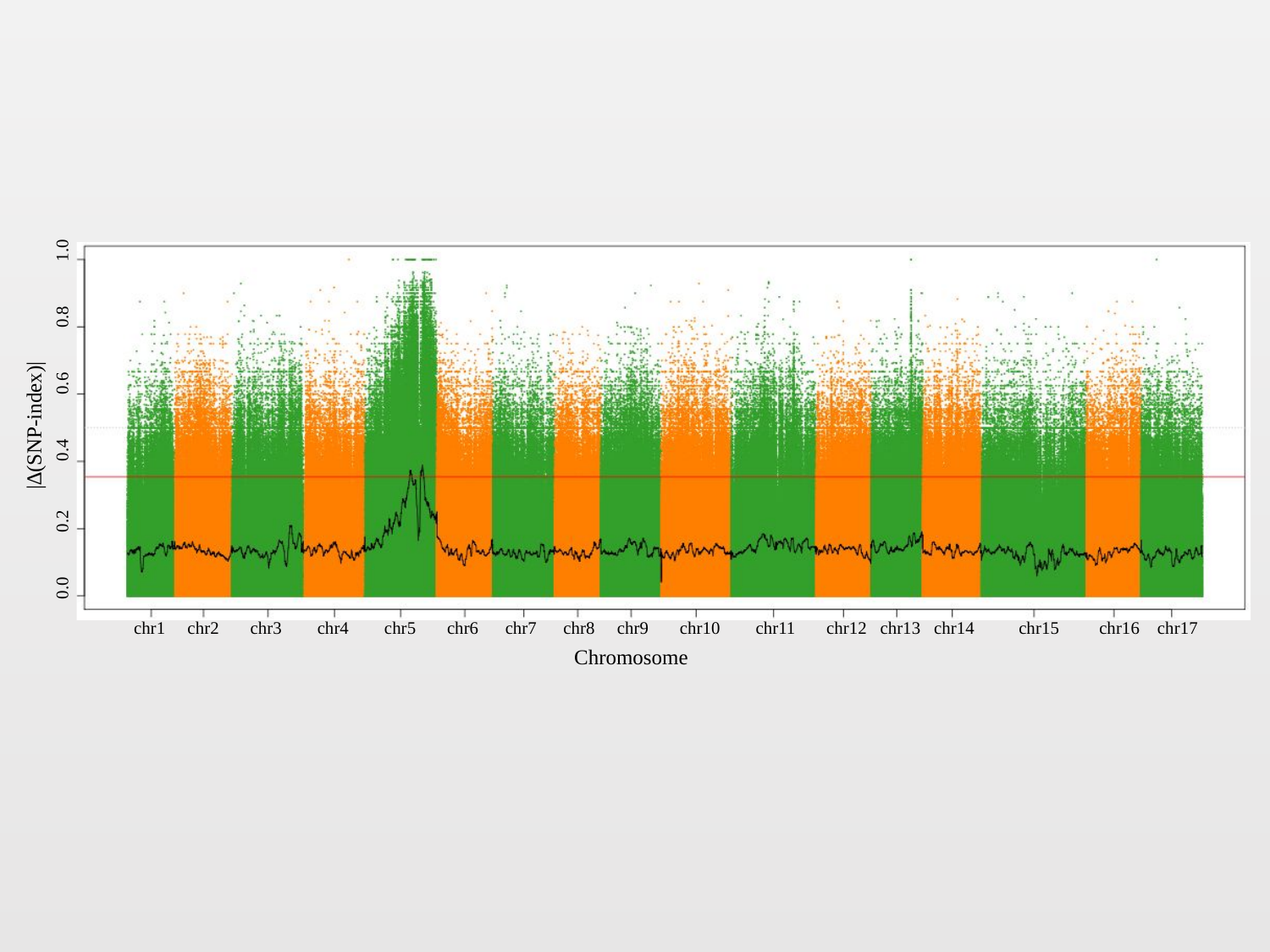

0.0 0.2 0.4 0.6 0.8 1.0
|∆(SNP-index)|
chr1 chr2 chr3 chr4 chr5 chr6 chr7 chr8 chr9 chr10 chr11 chr12 chr13 chr14 chr15 chr16 chr17
Chromosome

Supplement: Supplementary file 2 — Figure S1. |Δ(SNP-index)| graph derived from the modified QTL-seq analysis of mapping for the red skin trait of Asian pear. The X-axis indicates the position of the 17 pseudo-chromosomes and the Y-axis indicates the |Δ(SNP-index)|. (PPTX 278 kb) [file 12864_2018_5224_MOESM2_ESM.pptx]
